# Prevalence of symptomatic toxicities for novel therapies in adult oncology trials: a scoping review

**DOI:** 10.1093/jncics/pkaf036

**Published:** 2025-03-27

**Authors:** Amanda L King, Tamara Vasilj, Diane Cooper, Elizabeth Vera, Sefanit Berhanu, Morgan Johnson, Ciara Locke, Bennett Mciver, Ethan Basch, Joseph C Cappelleri, Amylou Dueck, Mark R Gilbert, Lee Jones, Yuelin Li, Lori M Minasian, Bryce B Reeve, Terri S Armstrong, Tito Mendoza

**Affiliations:** Office of Patient-Centered Outcomes Research, National Cancer Institute, Bethesda, MD, United States; Immune Deficiency Cellular Therapy Program, National Cancer Institute, Bethesda, MD, United States; National Institutes of Health Library, National Cancer Institute, Bethesda, MD, United States; Office of Patient-Centered Outcomes Research, National Cancer Institute, Bethesda, MD, United States; Office of Patient-Centered Outcomes Research, National Cancer Institute, Bethesda, MD, United States; Neuro-Oncology Branch, National Cancer Institute, Bethesda, MD, United States; Office of Patient-Centered Outcomes Research, National Cancer Institute, Bethesda, MD, United States; Neuro-Oncology Branch, National Cancer Institute, Bethesda, MD, United States; Division of Oncology, University of North Carolina—Chapel Hill, Chapel Hill, NC, United States; Statistical Research and Data Science Center, Pfizer Inc, Groton, CT, United States; Division of Health Sciences Research, Mayo Clinic, Rochester, MN, United States; Neuro-Oncology Branch, National Cancer Institute, Bethesda, MD, United States; Patient Advocate, United States; Department of Epidemiology and Biostatistics, Memorial Sloan Kettering Cancer Center, New York, NY, United States; Division of Cancer Prevention, National Cancer Institute, Bethesda, MD, United States; Department of Population Health Sciences, Duke University School of Medicine, Durham, NC, United States; Office of Patient-Centered Outcomes Research, National Cancer Institute, Bethesda, MD, United States; Neuro-Oncology Branch, National Cancer Institute, Bethesda, MD, United States; Office of Patient-Centered Outcomes Research, National Cancer Institute, Bethesda, MD, United States

## Abstract

**Background:**

Patients’ self-report of their symptoms can provide important data for the evaluation of treatment benefit and tolerability of oncology drugs. Contemporary treatment approaches, including immunotherapy and molecular targeted therapies, have unique toxicities based on their novel mechanisms of action. This scoping review aimed to summarize evidence from existing reviews and clinical practice guidelines to examine the type and prevalence of toxicities including symptomatic adverse events (sympAEs) for adult cancer patients to inform clinical care and therapeutic trials.

**Methods:**

A systematic search of PubMed, Web of Science, and Embase was performed using predefined eligibility criteria. Thirty-one literature reviews and 3 clinical practice guidelines met inclusion criteria and were selected for review and data abstraction.

**Results:**

Findings from this scoping review demonstrated several leading sympAEs that were reported across immunotherapy and targeted therapy drugs, including fatigue, diarrhea, and rash. In addition to these more prevalent sympAEs, there were some less frequently reported class-specific sympAEs, which had potential for significant harm or disability to the patient if not properly identified and treated. Many studies reported toxicities as AEs or syndromes solely using data reported by clinicians without additional self-report from patients.

**Conclusion:**

We identified several core sympAEs experienced by patients participating in oncology trials using immunotherapy and targeted therapy agents, which has implications for future trial design and drug labeling. Future cancer trials should assess patient-reported sympAEs based on the identified drug mechanism to inform the tolerability of these newer agents and enhance patient safety during trial participation and clinical care.

## Introduction

The US Food and Drug Administration (FDA) defines an adverse drug experience as any adverse event (AE) associated with the use of a drug in humans, regardless of whether they are considered drug related.^1^ For cancer clinical trials, the most widely used method clinicians use for reporting AEs is the Common Terminology Criteria for Adverse Events (CTCAE), with version 6.0 being the most current iteration of this measurement system.^2^ It has been well documented that clinicians can underestimate symptomatic toxicities (sympAEs) of patients under their care,^3-5^ which highlights the need for collection of self-reported symptoms using well-validated patient-reported outcomes measures (PROMs) to more accurately capture these important outcomes. In addition, assessing and monitoring sympAEs using PROMs as part of routine care has been shown to improve treatment adherence, tolerability, and survival.^6^^,^^7^ The National Cancer Institute (NCI) developed the Patient-Reported Outcomes version of the CTCAE (PRO-CTCAE) to facilitate the complementary reporting of symptomatic AEs directly from patients.^8^ Available to the public since 2014, the adult version of the PRO-CTCAE item library consists of 124 items representing 78 sympAEs,^9^ shown in Figure S1, with a pediatric version also available.^10^

Novel approaches to cancer treatment, such as immunotherapy and targeted therapies, provide an important paradigm shift in the field that is focused on exploiting genetic and molecular characteristics of the tumor or augmenting the immune system response. However, these newer cancer therapies are also associated with a unique spectrum of toxicities based on the nontraditional mechanisms of action,^11^ which may not be reflected in item libraries of previously developed PROMs.^12^ The aim of this scoping review is to summarize the prevalence and severity of the most common sympAEs, along with other toxicities, associated with newer cancer therapies for adult cancer patients based on evidence from published reviews and clinical practice guidelines to inform the timely and relevant selection of patient-reported sympAEs for incorporation into cancer clinical trials and care settings.

## Methods

### Search strategy

This scoping review was conducted according to the Preferred Reporting Items for Systematic Reviews and Meta-Analyses Extension for Scoping Reviews guidelines, as shown in Table S1. The population, intervention, comparison, and outcomes (PICO) framework^13^ was used to develop the following research question that guided this analysis: “For adult patients with cancer on novel oncological therapies, what are the most important (ie, common and severe) symptomatic adverse events that are reported?” A literature search of PubMed, EMBASE, and Web of Science was conducted by a research librarian (DC) on July 18, 2023, using a search strategy that included terms involving adverse events related to novel oncological agents, including immunotherapy and molecular targeted therapy. These search terms were combined with the term “neoplasms” to narrow the focus exclusively to cancer trials in the following search string: limits to English, 2014–present → (immunotherapy/ae OR molecular targeted therapy/ae OR precision medicine/ae) AND (neoplasms/dt). In addition to the systematic database search, we also examined the reference lists of the full-text articles that met our search criteria to identify additional relevant articles.

### Article selection and data extraction

The criteria for inclusion in this scoping review included the following: (1) review articles and clinical practice guidelines, (2) written in English language, (3) article published January 2014 through August 2023, 4) adult study population (≥18 years of age), (4) with a cancer diagnosis (all cancer types included), and (5) study participants were treated with novel oncological therapies on a clinical trial. We set the January 2014 criterion in our literature search because this year is when immune checkpoint inhibitors (ICIs) and many other novel cancer drugs began to be reported on in clinical trials in the United States. Additionally, 2014 is the year that the adult PRO-CTCAE validation study was published; therefore, the likelihood of trials using this instrument, in addition to the CTCAE, to report sympAEs would be more likely beyond that point.

**Figure 1.**
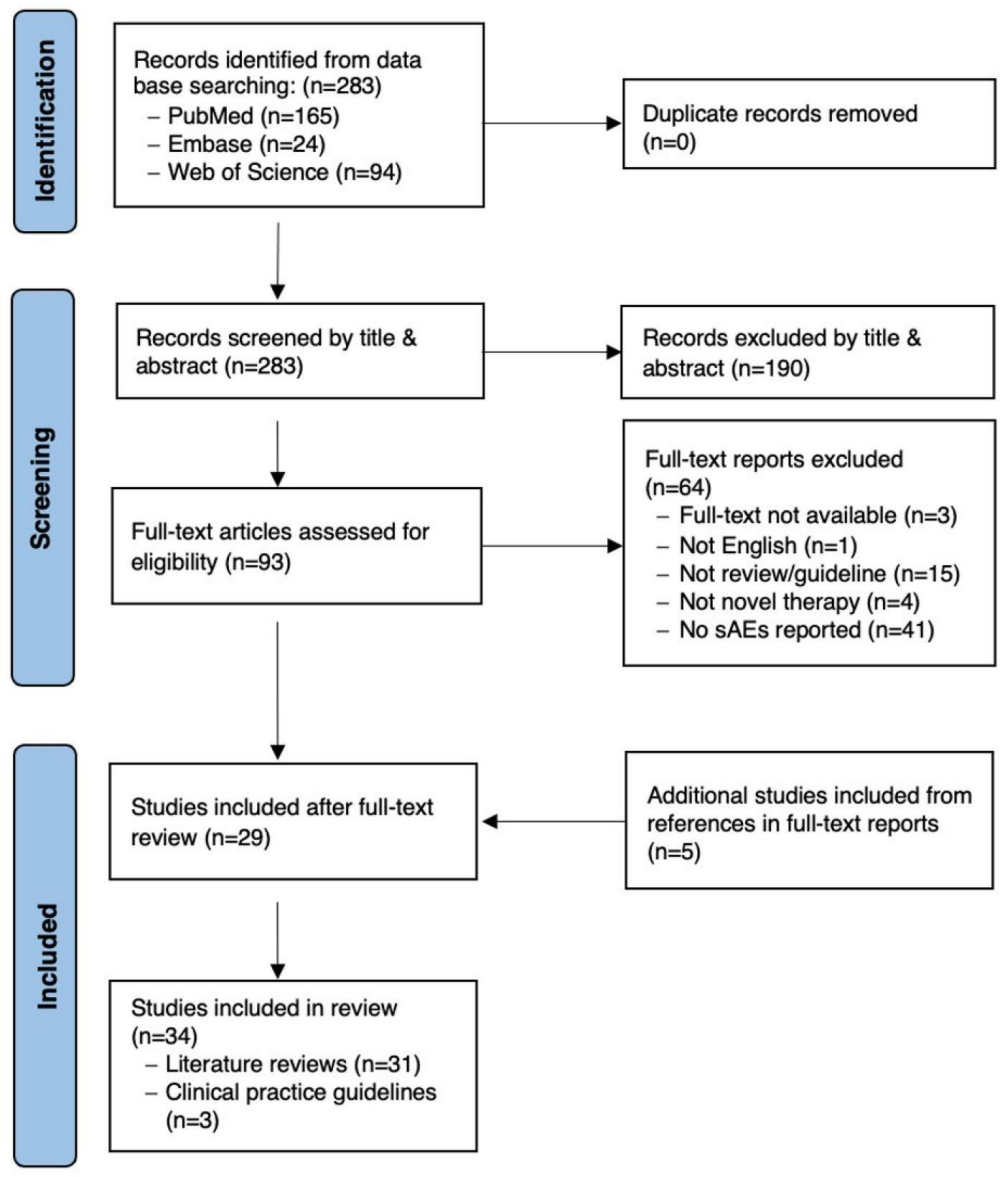
Scoping review consort diagram. Flow diagram for the scoping review of literature reporting on the prevalence of the most common symptomatic toxicities (sympAEs) associated with newer cancer therapies.

Covidence^14^ served as our primary tool for the data screening and extraction process. Two reviewers (ALK and TV) conducted title, abstract, and full-text screening for each article to determine their relevance and appropriateness for inclusion, with disputes resolved by a third reviewer (TSA or TM). Figure 1 outlines our search strategy. The initial literature search yielded 283 articles; of those, 254 articles were excluded because they did not meet the inclusion criteria after a review of the article. Twenty-nine articles were included for full-text review along with an additional 5 articles identified from reference lists of included studies, resulting in a total of 34 articles included in this review.

From relevant articles, data extraction was performed by ALK, TV, SB, MJ, CL, or BM and included information about the clinical trials in each review article when available (ie, trial phase), mechanism of action (MOA) of the study drug (different subtypes of immunotherapy or targeted therapy), study population tumor category (solid tumor, hematological, or mixed cancers) and tumor types, grade and incidence of sympAEs, as well as any other considerations deemed important in elucidating the AE profile of the study drug. When extracting data related to symptomatic toxicities, we referred to the PRO-CTCAE item library to categorize reported symptoms into existing item categories whenever possible and identify missing items that may warrant inclusion in future versions of the item library. Although our focus was on sympAEs, reported by either CTCAE or PRO-CTCAE, we documented all toxicities reported in the literature for context and reported in summary findings below.

### Statistical methods

We organized the prevalence of sympAEs by categorizing newer cancer therapies into 2 broad categories: immunotherapy and targeted therapy. Immunotherapy drugs include ICIs, T-cell therapies, immunomodulatory drugs, and cancer vaccines. Within the targeted therapy group, we further divided this category into small molecule therapies and monoclonal antibodies. In addition, for each of the 34 articles included in this scoping review, we noted the prevalence ranges for all grades of sympAEs. We report the range of prevalence rather than mean values because prevalence data from the review articles include sympAEs data from multiple trials, which may result in overlap among data reported across the included literature. Hence, we reported the minimum and maximum prevalence for all grades of the most prevalent sympAEs reported. If we do not report a range, it implies that the reviewed article(s) did not report a prevalence range and that there was only a single prevalence value for that specific sympAE, or if there was mention of various toxicities related to novel cancer drugs with no specific prevalence given. Figures 3 and 4 show the prevalence of sympAEs and other toxicities reported, based on drug category, with toxicities identified in at most 10% of patients shown. This prevalence threshold was chosen to highlight symptoms that are most likely to adversely affect trial participants.

## Results

### Included articles

Of the included 34 articles, the vast majority (82%) were topical reviews of clinical trials data, 3 articles (9%) were systematic reviews or meta-analyses (or both) of trials’ data, and 3 articles (9%) were clinical practice guidelines related to management of toxicities for patients taking immunotherapy or targeted therapy drugs. The clinical trials data were primarily from later-phase trials (ie, phases 2 and 3), although there were some early-phase sympAEs reported from phase 1 trials. Additionally, some articles reported toxicity data from clinical trials’ databases, including the Food and Drug Administration Adverse Events Reporting System (FAERS),^15^^,^^16^ the World Health Organization (WHO),^17^ and the French National Registry.^18^ Twenty-six of the 34 articles reported on at least 1 symptomatic toxicity related to use of a novel agent. Detailed information about the included studies in the review and reported drug toxicities by treatment type can be found in Table 1.

**Table 1.** Toxicities for novel cancer therapies by drug target.

Author (year)	Source info	Tumor population	Drugs and targets	**sympAEs** (% prevalence)	**AEs/Syndromes** (% prevalence)
***Immune Checkpoint Inhibitors***					
Bhandari (2018)	Meta-analysis of trials data 1 phase 2 trial 8 phase 3 trials	Mixed cancers (metastatic melanoma, RCC, lung, urothelial carcinoma, HNSCC, lymphoma)	**CTLA-4 inhibitors** (ipilimumab, tremelimumab) **PD-1/PD-L1 inhibitors** (nivolumab, pembrolizumab)	**GI:** diarrhea (11%-33%) **Skin:** pruritus (10%-35%), rash (12%-33%) **Sleep/Wake:** fatigue (15%-35%)	None reported
Brahmer (2019)	Systematic review of trials data ASCO Clinical Practice Guidelines for management of toxicities related to ICI therapy	Mixed cancers	**CTLA-4 inhibitor** (ipilimumab) **PD-1/PD-L1 inhibitors** (nivolumab, pembrolizumab, atezolimumab, durvalumab, avelumab)	**Oral:** dysphagia **GI:** N/V, cramping, constipation, diarrhea (19%-54%), abdominal pain	**GI:** colitis (8%-27%), intestinal perforation (1%), hepatitis (25%-30%)
Casaluce (2018)	Review of trials data 1 phase 2/3 trial 2 phase 3 trials 1 phase 3/4 trial	Solid tumor (NSCLC)	**PD-1/PD-L1 inhibitors** (nivolumab, pembrolizumab)	**GI:** nausea (9%-12%), diarrhea (7%-8%), ↓ appetite (10%-14%) **Skin:** rash (4%-9%) **Sleep/Wake:** fatigue (14%-16%), asthenia (6%-10%)	**Resp:** pneumonitis (3%-5%) **Endo:** hypothyroidism (4%-8%), hyperthyroidism (4%-6%)
Garcia (2019)	Review of FAERS database	Solid tumor (melanoma, NSCLC, pleural mesothelioma, RCC, colorectal)	**CTLA-4 inhibitor** (ipilimumab) **PD-1/PD-L1 inhibitors** (nivolumab, pembrolizumab, atezolimumab, avelumab)	**Neuro:** paresthesias (26%), hand/foot numbness (13%), extremity weakness (26%), ataxia (13%) **Visual:** vision changes (26%) **Sleep/Wake:** fatigue (26%) **GU:** loss of bladder control (13%) **Attention/Memory:** memory loss (13%), confusion (13%) **Misc:** fever (13%)	None reported
Gomatou (2020)	Review of trials data 2 phase 2 trials 4 phase 3 trials	Mixed cancers (NSCLC, melanoma, RCC, colorectal, urothelial carcinoma, Hodgkin lymphoma, head and neck, NSCLC, SCLC, MCC, MSI-H and MMRd, gastric, HCC, cervical, PMBCL, CSCC)	**CTLA-4 inhibitor** (ipilimumab) **PD-1/PD-L1 inhibitors** (nivolumab, pembrolizumab, atezolimumab, avelumab, durvalumab, cemiplimab)	None reported	**Resp:** pneumonitis (4%-19%)
Gordon (2017)	Review of trials data	Mixed cancers (bladder, melanoma, NSCLC, RCC, head and neck, Hodgkin lymphoma, MCC, SCLC)	**CTLA-4 inhibitor** (ipilimumab) **PD-1/PD-L1 inhibitors** (nivolumab, pembrolizumab, atezolimumab) **Combination therapy** (ipilimumab + pembrolizumab)	**GI:** diarrhea (2%-26%)	**Resp:** pneumonitis (1%-6%) **GI:** hepatitis (1%-13%) **Skin:** dermatitis (2%-23%) **Endo:** endocrinopathies (2%-39%)
Haanen (2022)	Systematic review of trials data ESMO Practice Guidelines for management of immunotherapy toxicities	Mixed cancers	**CTLA-4 inhibitor** (ipilimumab) **PD-1/PD-L1 inhibitors** (nivolumab, pembrolizumab, atezolimumab, avelumab, durvalumab)	**GI:** diarrhea (10%-35%), N/V, abdominal pain, hematemesis **Oral:** dysphagia, odynophagia **Resp:** dyspnea, cough, chest pain **Misc:** fever **Skin:** maculopapular rash (<5%), pruritus, lichenoid eruptions, psoriasis, skin depigmentation (5%-25%) **Neuro:** peripheral neuropathy (<1%), extremity weakness, ataxia **Vision:** ptosis, diplopia, dry or itchy eyes, changes in vision **Pain:** arthralgia (1%-43%), myalgia (2%-20%), joint stiffness and swelling	**GI:** hepatitis (5%-30%), pancreatitis (4%), colitis (10%-35%) **Resp:** pneumonitis/interstitial lung disease (2%-4%) **CV:** cardiac toxicity (<5%) **Skin:** cutaneous AEs (>50%) **Neuro:** neurotoxicity (1%-5%), myasthenia gravis-like syndrome **Vision:** ocular toxicity (<1%) **Pain:** inflammatory arthritis (5%-10%) **Rheum:** polymyalgia rheumatica syndrome (5%-10%) **Renal:** renal toxicity (2%-7%) **Endo:** primary hypothyroidism (4%-16%), hyperthyroidism (2%-10%), hypophysitis (1%-10%), type 1 DM (1%-2%), primary adrenal insufficiency (1%-8%) **Heme:** cytopenias
Helissey (2016)	Review of trials data 4 phase 3 trials	Solid tumor (NSCLC, melanoma)	**CTLA-4 inhibitors** (ipilimumab, tremelimumab) **PD-1/PD-L1 inhibitors** (nivolumab, pembrolizumab)	**GI:** diarrhea (>10%), anorexia (>10%), N/V (>10%), abdominal pain (>10%) **Skin:** rash (>10%), pruritus (>10%) **Pain:** arthralgia (>10%) **Sleep/Wake:** fatigue (>10%)	**GI:** dysimmune colitis **Resp:** pneumonitis
Kennedy (2020)	Review of trials data WHO database and multicenter trials registry	Mixed cancers	**CTLA-4 inhibitor** (ipilimumab) **PD-1/PD-L1 inhibitors** (nivolumab, pembrolizumab) **Combination therapy** (pembrolizumab + nivolumab, nivolumab + ipilimumab)	**GI:** diarrhea (27%-54%) **Skin:** rash (14%-17%)	**GI:** colitis (8%-22%), hepatic toxicities (17%) **Resp:** pneumonitis (5%) **Endo:** hypophysitis (1%-6%), hypothyroidism (22%), thyrotoxicosis (78%) **CV:** myocarditis (1%), pericarditis, arrhythmias
King-Kallimanis (2018)	Review of trials data	Solid tumor (NSCLC)	**PD-1/PD-L1 inhibitors**	**GI:** ↓ appetite (23%-25%), nausea (18%-20%), diarrhea (16%-19%) **Resp:** cough (26%-28%), dyspnea (24%-26%) **Skin:** rash (10%-11%), pruritus (9%-11%) **Pain:** MS pain (9%-13%) **Sleep/Wake:** fatigue (40%-49%) **Misc:** fever (12%-19%)	None reported
Lalani (2017)	Review of trials data 1 phase 1 trial 2 phase 1/2 trials 5 phase 2 trials 3 phase 3 trials	Solid tumor (prostate, urothelial carcinoma, RCC)	**CTLA-4 inhibitor** (ipilimumab) **PD-1/PD-L1 inhibitors** (nivolumab, pembrolizumab, atezolizumab, durvalumab, avelumab)	None reported	**Misc:** irAEs (2%-40%), trAEs (7%-37%)
Lyu (2022)	Review of trials data	Solid tumor (HCC)	**PD-1/PD-L1 inhibitors** (nivolumab, pembrolizumab, atezolizumab) **Combination therapy** (nivolumab + ipilimumab, atezolizumab + bevacizumab)	**GI:** ↓ appetite (1%), nausea (1%), diarrhea (1%-4%), constipation (1%), abdominal pain (1%) **Pain:** back pain (1%) **Skin:** pruritus (0%-4%), rash (0%-4%) **Sleep/Wake:** fatigue (2%-24%) **Misc:** fever (1%)	**Resp:** pneumonitis (1%-6%) **Skin:** serious skin reactions (2%) **CV:** HTN (15%) **GI:** transaminitis (4%-24%), hyperlipasemia (6%-13%), hyperamylasemia (4%), hyperbilirubinemia (2%-8%) **Heme:** anemia (2%-9%), thrombocytopenia (3%) **Endo:** adrenal insufficiency (2%) **Misc:** transfusion reactions (2%), trAEs (19%-73%)
Puzanov (2017)	Review of trials data & multidisciplinary workshop Society for Immunotherapy of Cancer Toxicity Management Working Group	Mixed cancers	**CTLA-4 inhibitor** (ipilimumab) **PD-1/PD-L1 inhibitors** (nivolumab, pembrolizumab, atezolimumab, avelumab, durvalumab)	**GI:** diarrhea (23%-44%) **Resp:** SOB, cough, wheezing, chest pain, ↓ exercise tolerance **CV:** palpitations, irregular heartbeat **Sleep/Wake:** fatigue, weakness **Skin:** maculopapular rash (30%-40%), pruritus (30%-40%), vitiligo (8%) **Neuro:** altered mental status, seizures **Vision:** blurred vision, diplopia, flashing lights, floaters, photophobia **Pain:** HA, arthralgia (15%)	**Renal:** renal AEs (2%-5%) **Heme:** hematological AEs (rare) **Endo:** acute hypophysitis (10%-17%), thyroid disorders (6%-20%), primary adrenal insufficiency, type 1 DM **GI:** hepatitis (4%-30%) **Resp:** pneumonitis (<5%) **Skin:** skin toxicities (30%-50%) **Neuro:** neurotoxicity (3%-6%), PRES **Vision:** ocular toxicity (<1%) **Misc:** infusion reactions (10%-25%)
Shingarev (2019)	Systematic review and meta-analysis of trials data	Mixed cancers	**CTLA-4 inhibitors** **PD-1/PD-L1 inhibitors**	**GI:** diarrhea (10%) **Skin:** rash, pruritus	**GI:** hepatitis **Resp:** pneumonitis **Renal:** renal failure (2%), AKI (5%) **Endo:** adrenal insufficiency
van Holstein (2019)	Review of trials data	Solid tumor (melanoma, NSCLC, bladder carcinoma, GI, gynecological, head and neck, breast, RCC, colorectal)	**CTLA-4 inhibitor** (ipilimumab) **PD-1/PD-L1 inhibitors** (nivolumab, pembrolizumab, atezolimumab) **Combination therapy** (ipilimumab + nivolumab)	**GI:** diarrhea (21%-30%) **Skin:** rash (39-40%), vitiligo (8%-10%)	**Endo:** endocrinopathies (53%) **GI:** hepatitis, colitis **Resp:** pneumopathy **Misc:** irAEs (13%-88%), trAEs (11%-87%)
Villani (2022)	Review of trials data 2 phase 2 trials 2 open-label, phase 3 trials	Solid tumor (CSCC, BCC)	**PD-1/PD-L1 inhibitors** (cemiplimab, pembrolizumab)	**GI:** constipation (15%),↓ appetite (15%), diarrhea (18%-27%) **Skin:** pruritus (18%-27%) **Sleep/Wake:** fatigue (15%-41%), asthenia (13%-21%)	None reported
***T-Cell Therapy***					
Al-Mansour (2022)	Review of trials data 3 phase 1 trials 1 phase 1 b trial 1 phase 1/2 trial 1 phase 2 trial 1 phase 3 trial	Hematological (R/R MCL and NHL)	**Anti-CD19 CAR-T cells** (axicabtagene, ciloleucel, lisocabtagene, maraleucel) **BiTE** (blinatumomab, mosenutuzumab, glofitamab, odronextamab)	None reported	**Neuro:** neurotoxicity (3%-71%) **Misc:** CRS (7%-90%)
Buitrago (2019)	Review of trials data 1 phase 1 trial 1 phase 1/2 trial	Hematological (R/R B lymphoid malignancies and B-ALL)	**Anti-CD19 CAR-T cells**	None reported	**Mood:** neuropsychiatric disorders (5%) **Immune:** infections (14%-42%), hypogammaglobinemia (41%), cytopenias (8%) **Misc:** subsequent malignancies (14%)
Chohan (2023)	Review of trials data 1 phase 1 trial 2 phase 1/2 trials 8 phase 2 trials 3 phase 3 trials 1 post-market surveillance study	Hematological (R/R B-ALL, DLBCL, PMBL, transformed FL, LBCL, MCL, multiple myeloma)	**Anti-CD19 CAR-T cells** (ciloleucel, autoleucel, tisagenlecleucel, brexucabtagene, axicabtagene, lisocabtagene, maraleucel) **Anti-BMCA CAR-T cells** (idecabtagene, vicleucel, ciltacabtagene, autoleucel)	None reported	**Neuro:** neurotoxicity (2%-64%) **Misc:** CRS (5%-95%)
Deshpande (2022)	Review of trials data 1 phase 1 1 phase 1/2 1 phase 2 1 multicenter safety study French National Registry trials data and retrospective records review	Hematological (R/R MCL and CLL)	**Anti-CD19 CAR-T cells** (autoleucel, brexucabtagene, axicabtagene, lisocabtagene, maraleucel) **Combination therapy** (anti-CD19 CAR-T cells + ibrutinib)	None reported	**Resp:** pneumonia (9%) **Neuro:** neurotoxicity (8%-63%) **Heme:** cytopenias (84%-94%) **Immune:** infections (32%) **Misc:** CRS (3%-91%)
El-Cheikh (2023)	Review of trial data 1 phase 1 b/2 trial	Hematological (R/R multiple myeloma)	**Anti-BMCA CAR-T cells** (clitacabtagene, autoleucel)	None reported	**CV:** HTN (6%)
Ganatra (2019)	Review of trials data 1 phase 1/2 trial 3 phase 2 trials	Hematological (R/R DLBCL and B-ALL)	**Anti-CD19 CAR-T cells** (ciloleucel, tisagenlecleucel, axicabtagene)	None reported	**CV:** hypotension (14%), cardiac arrest (1%-4%), tachycardia, QT prolongation, troponin elevation
Gutierrez (2018)	Systematic review of trials data and guidelines	Hematological (lymphoid malignancies)	**Anti-CD19 CAR-T cells**	**GI:** nausea, diarrhea (13%-14%), vomiting (60%-93%) **Skin:** macropapular rash **Pain:** myalgia, arthralgia, HA	**GI:** hepatic failure **Resp:** pulmonary CLS, ARDS, pulmonary edema **CV:** tachycardia, arrhythmias, hyper/hypotension, cardiogenic shock **Neuro:** neurotoxicity (40%-64%) **Renal:** acute kidney injury **Heme:** coagulopathy **Misc:** CRS
Huang (2023)	Review of trials data 2 phase 1 trials 1 phase 1/2 trial 2 phase 2 trials 1 phase 3 trial 1 real-world study	Hematological (R/R MCL, B-NHL, CLL, B lymphoid malignancies and DLBCL)	**Anti-CD19 CAR-T cells** (autoleucel, brexucabtagene, lisocabtagene, maraleucel) **Anti-CD20 CAR-T cells** (MB-106, ADI-001)	**Sleep/Wake:** fatigue, tiredness (15%) **Misc:** night sweats, fever (91%)	**CV:** tachycardia, arrhythmias, hypotension **Neuro:** encephalopathy, ICANS (29%) **Immune:** infection **Heme:** hematological toxicity (34%) **Misc:** injection site reaction, CRS (35%-100%)
Kennedy (2020)	Review of trials data and ASTCT Consensus Guidelines	Hematological (B lymphoid malignancies)	**Anti-CD19 CAR-T cells**	**Skin:** rash **Pain:** myalgias **Sleep/Wake:** fatigue **Misc:** fever	**GI:** hepatic dysfunction, hepatosplenomegaly, hyperferritinemia **CV:** circulatory collapse **Heme:** coagulopathy **Neuro:** ICANS **Misc:** CRS (35%-100%)
Kimble (2021)	Review of trial data 1 phase 3, multisite trial	Hematological (R/R B-ALL)	**BiTE** (blinatumomab)	None reported	**Neuro:** ICANS (10%) **Heme:** neutropenia **Immune:** infections (5%) **Misc:** CRS
Ronson (2016)	Review of trials data 2 phase 2 trials	Hematological (R/R ALL & B-ALL)	**Anti-CD19 CAR-T cells** **BiTE** (blinatumomab)	**GI:** nausea **Pain:** HA **Sleep/Wake:** fatigue	**Neuro:** neurotoxicity (50%), global encephalopathy, seizures, neuropathy **Heme:** neutropenia, anemia **Misc:** CRS
***Immunomodulators***					
Barrientos (2015)	Review of trial data 1 phase 3, randomized trial	Hematological (CLL)	**CRL4 ubiquitin E3 ligase inhibitor** (lenalidomide)	None reported	**Heme:** thrombotic events, thrombocytopenia **Misc:** risk of death
El-Cheikh (2023)	Review and meta-analysis of trials data 14 phase 3 trials FAERS database	Hematological (multiple myeloma)	**CRL4 ubiquitin E3 ligase inhibitors** (lenalidomide, thalidomide, pomalidomide) **Combination therapy** (lenalidomide or pomalidomide + dexamethasone)	None reported	**CV:** cardiotoxic events (27%), stroke (3%), myocardial infarction (2%), death related to cardiac events
Fraz (2019)	Review of trials data 2 phase 3 trials	Hematological (multiple myeloma)	**CRL4 ubiquitin E3 ligase inhibitor** (lenalidomide)	**Neuro:** peripheral neuropathy	**Misc:** any AEs (80%-89%)
Lalani (2017)	Review of trials data 2 phase 2 trials 4 phase 3 trials	Mixed cancers	**Cytokines** (IFN, IL-2) **Combination therapy** (IFN + bevacizumab, IFN + tesirolimus)	None reported	**CV:** cardiac toxicity (44%) **Misc:** trAEs (11%-80%), death related to treatment (4%)
Romancik (2020)	Review of trials data 2 phase 2 trials	Hematological (MCL)	**CRL4 ubiquitin E3 ligase inhibitor** (lenalidomide) **Combination therapy** (lenalidomide + rituximab)	**Neuro:** peripheral neuropathy (21%)	**Heme:** hematological toxicity, myelosuppression, VTE, neutropenia (50%), thrombocytopenia (13%), anemia (11%) **Misc:** fetal toxicity
***VACCINES***					
Lalani (2017)	Review of trials data 1 phase 2 trial 1 phase 3 trial	Solid tumors (prostate, RCC, urothelial)	**PSA** (poxviral vaccine) **Combination therapy** (MVA-5T4 + IFN + IL-2, sunitinib)	**GI:** nausea **Skin:** erythema and itching **Neuro:** dizziness **Pain:** pain **Sleep/Wake:** fatigue **Misc:** fever, chills	**Misc:** trAEs (16%-57%)
***Small Molecule Therapies***					
Al-Mansour (2022)	Review of trials data Pooled trials analysis Retrospective analysis of phase 2/3 trials 1 phase 1 trial 1 phase 1/2 trial 4 phase 2 trials	Hematological (MCL)	**BTK inhibitors** (ibrutinib, acalabrutinib, zanubrutinib) **Proteosome inhibitors** (bortezomib, carfilzomib) **Combination therapy** (ibrutinib + rituximab, ibrutinib + bortezomib)	**GI:** diarrhea (37%), nausea (22%), constipation **Resp:** cough (23%) **Pain:** HA (39%), myalgia (22%)	**CV:** A-fib (2%-12%), HTN (3%-12%), acute coronary syndrome (2%), cardiorespiratory arrest (1%) **Resp:** pneumonia (≥5%) **Neuro:** neurotoxicity (15%) **Heme:** bleeding events (5%-10%), severe hemorrhage (3%-5%), neutropenia (≥5%), petechiae (≥5%), thrombocytopenia (≥5%), anemia (≥5%)
Barrientos (2015)	Review of trials data 2 phase 3 trials	Hematological (CLL)	**BTK inhibitor** (ibrutinib) **PI3K inhibitor combination therapy** (idelalisib + rituximab)	**GI:** diarrhea (14%) **Sleep/Wake:** fatigue **Misc:** fever	**GI:** colitis, transaminitis, colitis (14%) **Immune:** infections **Heme:** bleeding events
Belgioia (2019)	Review of trials data 13 phase 2 trials 1 phase 3 randomized trial	Solid tumor (NSCLC, esophageal SCC, SCCHN, LA-HNSCC, brain metastases, renal, HCC	**TK inhibitors** (gefitinib, erlotinib) **Combination therapy** (gefitinib + RT, erlotinib + RT, erlotinib + cisplatin + RT, erlotinib + docetaxel + RT, whole-brain RT + erlotinib/gefitinib, sunitinib + RT, sorafenib + RT)	**Oral:** dysphagia, mucositis **GI:** diarrhea (8%-9%) **Skin:** rash (4%-68%) **Sleep/Wake:** fatigue **Pain:** pain exacerbation (17%)	**Oral:** esophagitis (5%-28%) **Skin:** dermatitis **Resp:** pneumonitis (4%-5%), hypoxia (4%), ARDS (4%), interstitial pneumonia (8%) **CV:** HTN (5%) **Heme:** neutropenia (10%), anemia (10%) **Ortho:** vertebral body compression (4%), fractures (21%) **Misc:** AEs ≥3 (65%), any AEs (92%-94%)
El-Cheikh (2023)	Systematic review and meta-analysis of trials data FAERS database	Hematological (multiple myeloma)	**Proteosome inhibitors** (bortezomib, carfilzomib, ixazomib)	None reported	**CV:** cardiac AEs (18%), HF (1%-5%), HTN (3%), arrhythmias (1%-4%), acute MI (15%), ischemic heart disease (1%-6%), cardiac death (1%)
Fraz (2019)	Review of trials data	Hematological (multiple myeloma)	**Proteosome inhibitors** (bortezomib, carfilzomib, ixazomib)	**Resp:** dyspnea (3%) **Neuro:** peripheral neuropathy (2%-15%)	**CV:** cardiotoxicity, HTN (3%-25%), cardiac event (12%), cardiac failure (3%-20%)
Radhakrishnan (2021)	Review of trials data 4 phase 1 trials 1 phase 1/1 b trial 1 phase 1 b trial 7 phase 2 trials Pooled trials analysis	Hematological (MCL)	**BTK inhibitors** (ibrutinib, acalabrutinib, zanubrutinib) **Combination therapy** (acalabrutinib + rituximab + bortezomib, ibrutinib + bendamustine + rituximab, ibrutinib + palbociclib, ibrutinib + rituximab, ibrutinib + rituximab + lenalidomide, ibrutinib + venetoclax)	**GI:** N/V (71%), diarrhea (7%-38%) **Skin:** rash (4%-17%), hives **Pain:** HA, joint pain (42%) **Sleep/Wake:** fatigue (75%)	**GI:** hepatobiliary disorders **Resp:** pneumonia, pneumonitis (10%) **CV:** HTN, hypokalemia **Heme:** hematological toxicity (3%-11%), cytopenias, bleeding (9%) **Skin:** cutaneous reactions **Immune:** infection (11%)
Rogiers (2015)	Review of trials data 1 phase 1 trial 2 phase 3 trials	Solid tumor (melanoma)	**BRAF inhibitors** (dabrafenib, vemurafinib) **MEK inhibitor** (trametinib)	**GI:** diarrhea (43%) **CV:** peripheral edema (26%) **Skin:** rash (50%), photosensitivity (31%) **Pain:** joint pain (39%) **Sleep/Wake:** fatigue (26%-34%) **Misc:** fever	**CV:** HTN (40%-64%) **Skin:** hyperproliferative skin lesions (15%-30%)
Romancik (2020)	Review of trials data 1 phase 1 trial 8 phase 2 trials 2 phase 3 trials	Hematological (MCL)	**BCL-2 inhibitor** (venetoclax) **TK inhibitors** (entospletinib, zanabrutinib, ibrutinib, acalabrutinib) **Proteosome inhibitor** (bortezomib) **Combination therapy** (bortezomib + CHOP, bortezomib + rituximab + cyclophosphamide + doxorubicin + prednisolone, bortezomib + rituximab + bendamustine + dexamethasone)	**GI:** diarrhea (54%), nausea, constipation **Neuro:** peripheral neuropathy (4%-30%) **Pain:** HA **Sleep/Wake:** fatigue (18%) **Misc:** nosebleeds (50%)	**GI:** GI disturbances, transaminitis (15%) **Neuro:** neuropathy (15%) **Resp:** URIs, pneumonia **Heme:** myelosuppression, bleeding (6%), neutropenia (9%), thrombocytopenia (35%) **Immune:** infections
Santoni (2018)	Review of trials data 1 phase 1 b trial 1 phase 2 trial 1 phase 3 trial	Solid tumor (RCC)	**VEGFR TK inhibitor** (tivozanib) **Combination therapy** (tivozanib + temsirolimus)	**Oral:** dysphonia (21%) **Sleep/Wake:** fatigue (15%)	**CV:** HTN (12%-44%) **Heme:** thrombocytopenia (15%)
Villani (2022)	Review of trials data 5 phase 1 trials 4 phase 2 trials 1 phase 3 trial	Solid tumor (BCC)	**HH inhibitors** (vismodegib, sonidegib, patidegib, taladegib)	**GI:** dysgeusia (38%-68%), nausea (33%-47%), vomiting (32%), diarrhea (24%), ↓ appetite (19%-63%) **Pain:** HA (15%), myalgia (19%), arthralgia (13%) **Sleep/Wake:** fatigue (29%-54%), asthenia (3%) **Skin:** alopecia (38%-66%) **Misc:** muscle spasms (23%-71%), weight loss (9%-27%)	**GI:** ↑ lipase (5%) **CV:** HTN (3%) **Misc:** ↑ CK levels (3%-29%)
***Monoclonal Antibodies***					
Barrientos (2015)	Review of trials data Pooled trials data 3 phase 3 trials	Hematological (CLL)	**Anti-CD20** (rituximab) **Anti-CD52** (alemtuzumab) **Combination therapy** (rituximab + ofatumumab + obinutuzumab, rituximab + fludarabine + cyclophosphamide, rituximab + idelalisib)	**GI:** diarrhea **Misc:** fever	**GI:** transaminitis **Heme:** pancytopenia **Immune:** viral hepatitis and CMV reactivation, severe viral or fungal infections **Misc:** severe or life-threatening infusion reactions, treatment-related mortality (4%)
Belgioia (2019)	Review of trials data 23 trials (phases 1 and 2)	Solid tumor (GBM, head and neck, esophageal, bladder, SCCHN, rectal)	**HER2 inhibitor combination therapy** (trastuzumab + RT, trastuzumab + paclitaxel + RT, cetuximab + RT) **VEGF-A inhibitor combination therapy** (bevacizumab + RT)	**GI:** mucositis (31%)	**Oral:** esophagitis (5%) **CV:** cardiac toxicity **Skin:** skin toxicity, skin reactions (1%-4%) **Heme:** bleeding episodes (17%) **Immune:** delayed healing (7%), pelvic infections (14%) **Misc:** allergic reactions (6%), trAEs (35%)
El-Cheikh (2023)	Review of trials data 4 trials (phases 2 and 3)	Hematological (multiple myeloma)	**Anti-CD38 combination therapy** (daratumumab + carfilzomib + dexamethasone, isatuximab + dexamethasone)	**Resp:** dyspnea (15%-17%)	**CV:** cardiac AEs (29%), cardiac failure (7%-8%), ischemic heart disease (4%-5%), HTN (5%-20%)
Richardson (2020)	Review of trials data 2 phase 1 b trials 3 phase 1/2 trials 2 phase 3 trials	Hematological (multiple myeloma)	**Anti-CD38** (isatuximab) **Anti-CD38 combination therapy** (isatuximab + pomalidomide + dexamethasone, isatuximab + lenolidamide, isatuximab + carfilzomib + dexamethasone)	**GI:** nausea (35%-42%), diarrhea (26%-53%) **Resp:** cough (23%-40%) **Sleep/Wake:** fatigue (37%-64%)	**Resp:** respiratory infections (32%), URIs (24%-40%), bronchitis (24%), pneumonia (20%) **CV:** cardiac failure (4%) **Heme:** neutropenia (12%-96%), thrombocytopenia (30%-84%), anemia (99%), lymphopenia (34%) **Misc:** infusion reactions (3%-51%), overall AEs (77%-99%), serious AEs (59%), fatal AEs (3%)
Romancik (2020)	Review of trials data 5 phase 2 trials 3 phase 3 trials	Hematological (MCL)	**Anti-CD38** (isatuximab) **Anti-CD20 combination therapy** (rituximab + bendamustine, rituximab + cyclophosphamide + doxorubicin + vincristine + prednisone, rituximab + bendamustine + cytarabine)	**GI:** nausea **Skin:** alopecia **Neuro:** peripheral neuropathy (7%-29%) **Sleep/Wake:** fatigue	**Heme:** myelosuppression, thrombocytopenia (87%), neutropenia (12%) **Immune:** infections, viral reactivation **Misc:** erythematous skin reaction, infusion reactions
Ronson (2016)	Review of trials data 3 phase 2 trials	Hematological (ALL)	**Anti-CD22 combination therapy** (inotuzumab + ozogamicin)	None reported	**Heme:** veno-occlusive disease (23%)

Abbreviations: AEs = adverse events; ALL = acute lymphoblastic leukemia; ASCO = American Society of Clinical Oncology; ASTCT = American Society for Transplantation and Cellular Therapy; B-ALL = B-cell acute lymphoblastic leukemia; BCC = basal cell carcinoma; CAR-T = chimeric antigen receptor therapy; CLL = chronic lymphocytic leukemia; CLS = capillary leak syndrome; CRS = cytokine release syndrome; CSCC = cutaneous squamous cell carcinoma; DLBCL = diffuse large B-cell lymphoma; ESMO = European Society for Medical Oncology; FAERS = FDA Adverse Events Reporting System; FL = follicular lymphoma; GBM = glioblastoma; HA = headache; HCC = hepatocellular carcinoma; HNSCC = head and neck squamous cell carcinoma; ICANS = immune effector cell associated neurotoxicity; irAEs = immune-related AEs; LA = locally advanced; MCC = Merkel cell carcinoma; MCL = mantle cell lymphoma; MMRd = mismatch-repair deficient; MS = musculoskeletal; MSI-H = high microsatellite instability; N/V = nausea/vomiting; NHL = non-Hodgkin lymphoma; NSCLC = non-small cell lung cancer; PMBCL = primary mediastinal large B cell lymphoma; R/R = recurrent/refractory; RCC = renal cell carcinoma; SCC = squamous cell carcinoma; SCCHN = squamous cell carcinoma of head and neck; SCLC = small cell lung cancer; sympAEs = symptomatic adverse events; trAEs = treatment-related AEs; WHO = World Health Organization.

### Types of novel agents

**Figure 2.**
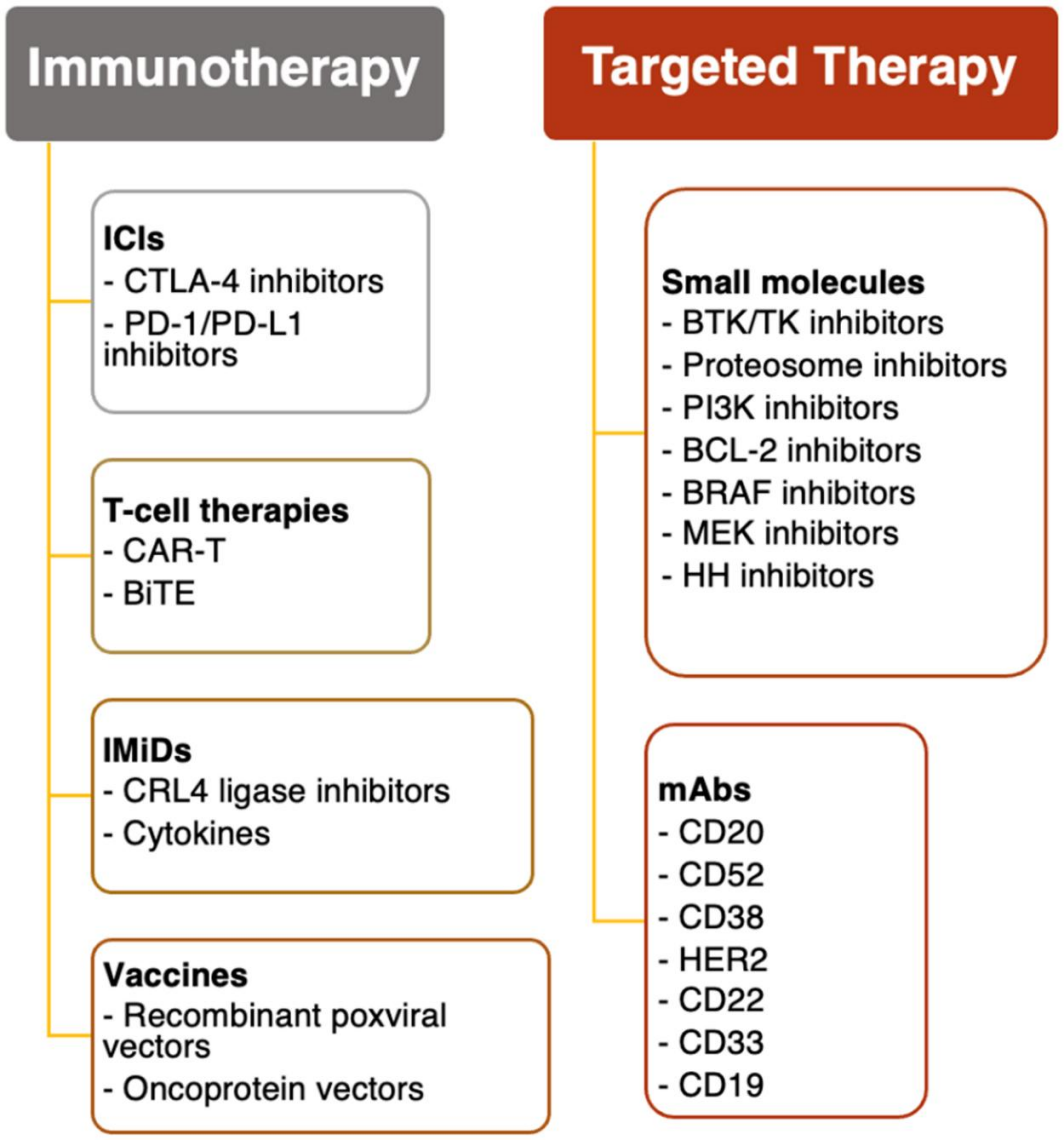
Classes of novel oncological agents examined in review. Two main categories of drugs are covered in this review with respect to their symptomatic toxicities (sympAEs): immunotherapy and targeted therapy. The immunotherapy category included immune checkpoint inhibitors (ICIs), T-cell therapies, immunomodulators (IMiDs), and cancer vaccines. The targeted therapy drugs included small molecule agents and monoclonal antibodies (mAbs). Targets for all drugs included in this review are listed under their respective drug classes.

Figure 2 details the MOA of the novel cancer drugs identified in this review, which are organized by the class of therapy. The majority of novel agents included were categorized as immunotherapy, which included a variety of different mechanisms. Immune checkpoint inhibitors work by blocking proteins on T-cells or cancer cells that typically stop the immune system from attacking cancer cells,^19^ which facilitates an enhanced antitumor response. This review presents 2 kinds of ICIs: CTLA-4 inhibitors^15^^,^^17^^,^^19-28^ and PD-1 inhibitors/PD-L1 inhibitors.^15^^,^^17^^,^^19-32^ T-cell therapy agents included chimeric antigen receptor T-cell (CAR T) therapy^16-18^^,^^33-39^ and bispecific T-cell engager (BiTE) therapy,^33^^,^^39^^,^^40^ both of which facilitate T-cell mediated killing of malignant cells.^41^ Immunomodulators, including cullin-RING E3 ubiquitin (CRL4) ligase inhibitors^16^^,^^42-44^ and cytokines,^25^ are drugs that modify the immune system by increasing or decreasing its response in order to help destroy cancer cells.^45^ Cancer vaccines, which are sometimes made with cells, peptides, or proteins from the patient’s own tumor, stimulate the immune system so it can recognize the cancer cells as foreign and encourage destruction of the tumor.^46^ Two kinds of cancer vaccines that are represented in this review were discussed in a single article:^25^ those using a recombinant poxviral vaccine vector and those using a fetal oncoprotein vector.

The other broad class of novel agents were classified as targeted therapy and included small molecules and monoclonal antibodies (mAbs). Because of their small size (typically ≤500 Da), small molecule drugs have been used successfully to target extracellular, cell surface ligand-binding receptors and the intracellular proteins that play important roles for cancer cell survival, proliferation, and metastasis.^47^ Several small molecule cancer drugs are represented in this review, including tyrosine kinase (TK) inhibitors,^33^^,^^42^^,^^44^^,^^48-50^ proteosome inhibitors,^16^^,^^33^^,^^43^^,^^44^ phosphoinositide 3-kinase (PI3K) inhibitors,^42^ B-cell lymphoma-2 (BCL-2) inhibitors,^44^ protein kinase B-raf (BRAF) inhibitors,^51^ mitogen-activated protein kinase (MEK) inhibitors,^51^ and Hedgehog pathways (HH) inhibitors.^32^ The other class of targeted therapy cancer drugs in this review is the mAbs,^16^^,^^39^^,^^42^^,^^44^^,^^48^^,^^52^ which are laboratory-designed mimics of naturally occurring antibodies that are created to recognize and find specific proteins on cancer cells in order to kill them.^53^ Some ICIs can also be considered mAbs, so for the purposes of this review the novel agents classified as mAbs are non-ICI mAbs.

### Main findings

**Figure 3.**
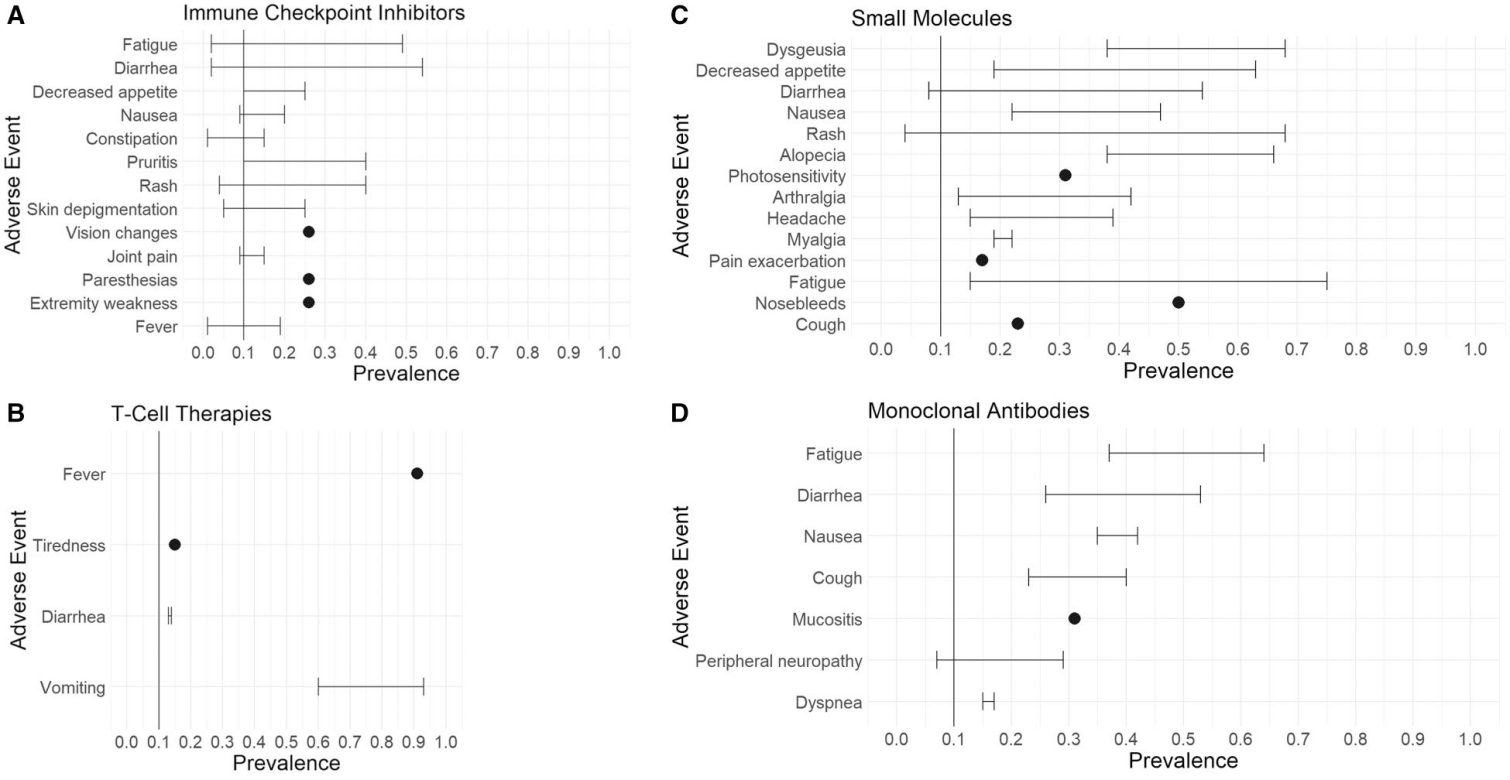
Most prevalent symptomatic AEs (all grades) for novel cancer therapies by drug target. Symptom prevalence ranges and point estimates are reported for immunotherapy drugs including immune checkpoint inhibitors (**A**) and T-cell therapies (**B**), and targeted therapy drugs including small molecules (**C**) and monoclonal antibodies (**D**), showing those reported in ≥10% of patients.

In the sections below, we have summarized the most commonly reported sympAEs and also for context other clinician-reported AEs or syndromes for each novel agent drug class. Additional details about novel drug toxicities and their source data can be found in Table 1. A summary of the most prevalent sympAEs for novel cancer therapies based on drug target is shown in Figure 3.

#### Immunotherapy

*Immune checkpoint inhibitors.* Twelve articles reported on toxicities related to ICIs, including 3 clinical practice guidelines. Immune checkpoint inhibitors were used in trials across all various cancer populations, including solid tumors and hematological cancers; the most common tumor types were lung,^15^^,^^20-22^^,^^24^^,^^28-30^ skin,^15^^,^^20-22^^,^^24^^,^^28^^,^^32^ renal,^15^^,^^20-22^^,^^25^ head and neck,^20-22^^,^^28^ and colorectal cancer.^15^^,^^21^^,^^28^ In the ICI articles where details about the clinical trials were provided, there was a stronger weight toward later-phase trials, with 23 phase 3 trials, 10 phase 2 trials, and 1 phase 1 trial reporting toxicity data. Additionally, some studies reported toxicities data from clinical trials databases, including FAERs,^15^ WHO,^17^ and a multicenter trials registry,^17^ which likely contain some of the same toxicity data reported in other articles. The clinical practice guidelines toxicity articles were put forth by medical organizations, such as the American Society for Clinical Oncology,^19^ the European Society for Medical Oncology,^23^ and the Society for Immunotherapy of Cancer,^26^ and included a systematic review of the clinical trials literature and recommendations for management of these toxicities.

**Figure 4.**
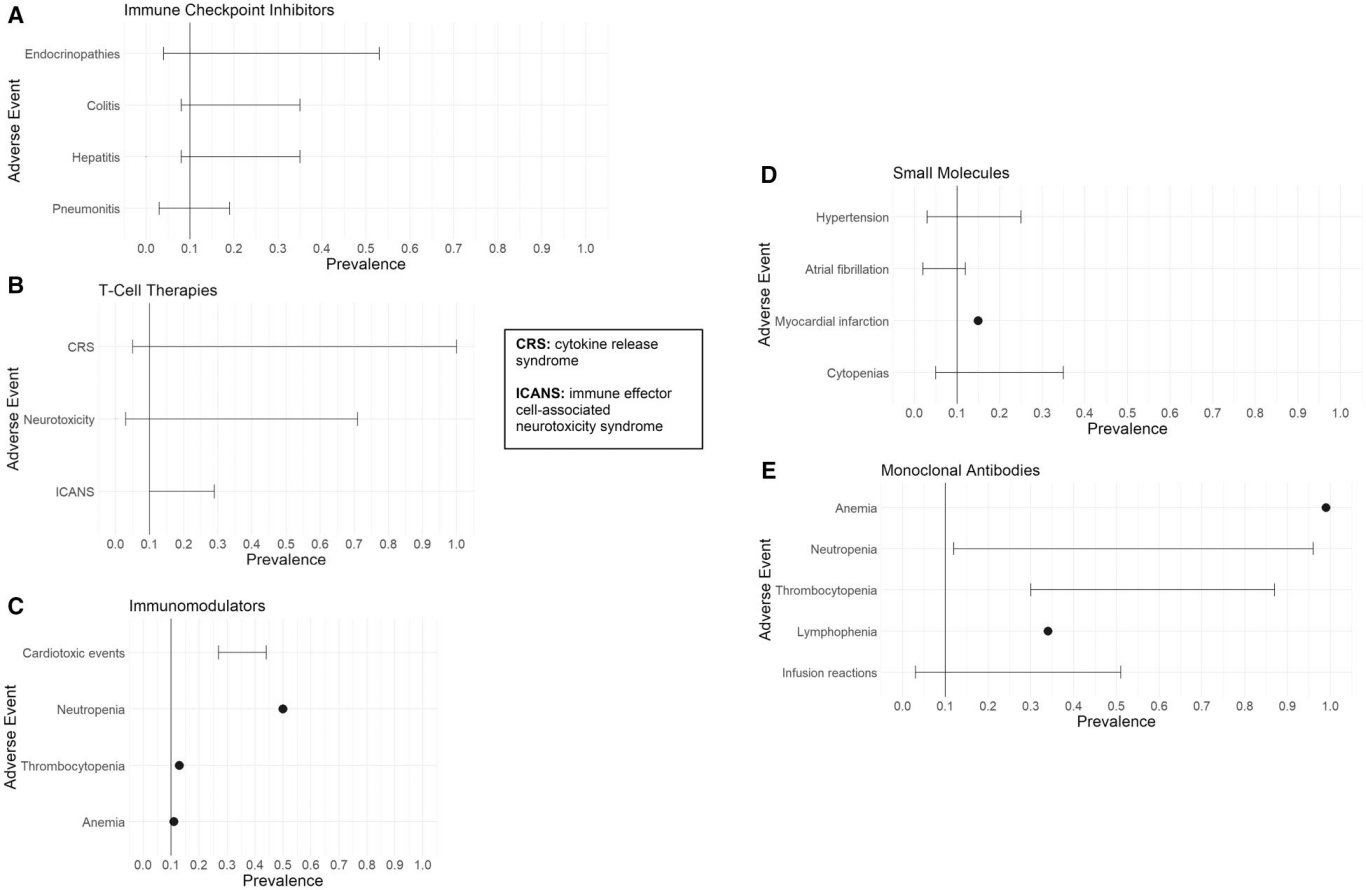
Most prevalent clinician-reported AEs (all grades) for novel oncological therapies by drug target. Toxicity prevalence ranges and point estimates are reported for immunotherapy drugs including immune checkpoint inhibitors (**A**), T-cell therapies (**B**), and immunomodulators (**C**), and targeted therapy drugs including small molecules (**D**) and monoclonal antibodies (**E**), showing those reported in ≥10% of patients.

Of the 12 articles reporting ICI toxicities, all but 1 article^21^ provided information about sympAEs and most reported additional AEs related to the drug therapy. The most common sympAEs related to ICI therapy were related to gastrointestinal (GI) and skin symptoms, shown in Figure 3, A. The most prevalent GI symptoms were diarrhea (2%-54%), decreased appetite (10%-25%), nausea (9%-20%), and constipation (1%-15%). Skin toxicities were very common with these agents but typically were low-grade (ie, grade 1 or 2).^23^ Commonly reported sympAEs related to skin included pruritus (10%-40%), rash (typically macropapular, 4%-40%), and skin depigmentation (5%-25%). Other frequently reported sympAEs related to ICI therapy included fatigue (2%-49%), vision changes (up to 26%), joint pain (9%-15%), paresthesias (26%), extremity weakness (26%), and fever (1%-19%). Figure 4, A shows other clinician-reported AEs that were commonly reported across the ICI studies, including endocrinopathies (4%-53%), colitis (8%-35%), hepatitis (4%-30%), and pneumonitis (3%-19%) with rare cardiac toxicities (<5%), which are often identified and characterized by their associated symptoms.

*T-cell therapies.* Eleven articles reported toxicities related to T-cell therapies, all of which were in hematological cancer populations. The most common hematological cancers represented in the included literature were B-cell acute lymphoblastic leukemia (B-ALL),^34-36^^,^^39^^,^^40^ mantle cell lymphoma (MCL),^18^^,^^33^^,^^35^^,^^38^ multiple myeloma (MM),^16^^,^^35^ diffuse large B-cell lymphoma (DLBCL),^35^^,^^36^^,^^38^ and chronic lymphocytic leukemia (CLL).^18^^,^^38^ In the T-cell therapy articles where details about the clinical trials were provided, there was a stronger weight toward providing data from earlier-phase trials, with 17 phase 1 or phase 1/2 trials, 18 phase 2 trials, and 6 phase 3 trials reporting toxicity data. Additionally, 1 study reported toxicity data from a French national clinical trials database,^18^ and another included trials data from the American Society for Transplantation and Cellular Therapy Consensus Guidelines.^17^

Of the 11 articles reporting T-cell therapy toxicities, only 4 (36%) reported any sympAEs from clinical trials using these agents, and few prevalence data were provided. The most common sympAEs related to T-cell therapy (shown in Figure 3, B) were fever (91%) and GI symptoms, including vomiting (60%-93%) and diarrhea (13%-14%), with nausea also mentioned without prevalence metrics. Additionally, tiredness (15%) was a sleep/wake disturbance reported by patients taking these drugs. Other sympAEs that were commonly associated with T-cell therapies, but lacked prevalence data in the literature, included fatigue, rash, headache, myalgia and arthralgia, and night sweats.

The vast proportion of clinician-reported AEs data reported about T-cell therapies were related to cytokine release syndrome (CRS; 5%-100%), neurotoxicity (3%-71%), and immune effector-cell associated neurotoxicity syndrome (ICANS; 10%-29%), as shown in Figure 4, B. The way these AEs were reported in the literature was by identifying the prevalence of these syndromes and providing which symptoms characterize them, rather than reporting prevalence data for the individual symptoms. Cytokine release syndrome was the most common acute AE after CAR T-cell therapy, and it was reported that the neurotoxicity typically occurs soon after CAR-T administration.^39^  Table 2 shows individual symptoms associated with common AE syndromes associated with T-cell therapy, as well as reported prevalence metrics based on AE grade. Other reported AEs related to T-cell therapy included cytopenias (8%-94%), infections (14%-42%), and subsequent malignancies (14%).

**Table 2.** T-cell therapy frequently reported adverse events and syndromes with grading metrics.

Adverse Events	Grade	Prevalence, %
***Cytokine release syndrome*** Includes fever, malaise, fatigue, nausea, vomiting, myalgia, and arthralgia	All grades	5-100
	Grades 1-2	50-74
	Grades ≥3	1-99
	Grades 4-5	3
***Neurotoxicity***	All grades	3-71
Includes tremors, encephalopathy, confusion, lack of attention, distant gaze, facial twitching, aphasia, coma, agitation, headache, nausea, and vomiting	Grades ≥3	2-35
***Immune effector-cell associated neurotoxicity syndrome***	Grades ≥3	10-29
Includes tremor, dysgraphia, aphasia, apraxia, impaired attention, and akinetic seizures		

*Immunomodulators.* Five articles reported toxicities related to immunomodulator (IMiD) therapies, which were used primarily in hematological cancers, such as MM,^16^^,^^43^ CLL,^42^ and MCL,^44^ although there were data from 1 trial with a mixed population of hematological cancers and solid tumors.^25^ In the IMiD articles where details about the clinical trials were provided, the reporting was weighted toward late-phase trials with 21 phase 3 trials and 4 phase 2 trials reporting toxicity data. Additionally, 1 study reported toxicity data from the FAERS clinical trials database.^16^

Of the 5 articles reporting IMiD therapy toxicities, only 2 (40%) reported any sympAEs from clinical trials using these drugs, and the only sympAE reported was peripheral neuropathy (21%). The majority of clinician-reported AE reporting from IMiD trials was related to significant cardiac and hematological toxicities (shown in Figure 4, C), some of which resulted in patient death. The reported prevalence of cardiotoxic events related to IMiDs was 27%-44%, which included myocardial infarction, stroke, and death. Hematological toxicities were also quite common, including neutropenia (50%), thrombocytopenia (13%), and anemia (11%). Myelosuppression and thrombotic events were also reported, but without prevalence data. The prevalence of treatment-related AEs (trAEs) for IMiDs was 11%-80% and the risk of death related to treatment was 4%, with identified risk factors including older age^25^ and concurrent use of other immunotherapy drugs.^16^

*Vaccines.* Only 1 article reported sympAEs related to cancer vaccine therapy.^25^ In this review, the authors discussed the use of 2 different vaccines in clinical trials (1 phase 1 trial, 1 phase 2 trial) for patients with solid tumors, including prostate cancer, renal cell carcinoma, and urothelial cancer. There were no specific prevalence data provided in this review article for sympAEs, but they reported that cancer vaccines can cause symptoms such as nausea, skin erythema and pruritus, dizziness, pain, fatigue, chills, and fever. Additionally, the authors provided an overall prevalence for trAEs of 16%-57% for these cancer vaccines.

#### Targeted therapy

*Small molecules.* Ten articles reported toxicities related to small molecule drugs, which were used in trials for both solid tumor and hematological cancers. Use in hematological cancers was more common (6 of 10 articles), with the most represented patient diagnoses including MCL,^33^^,^^44^^,^^49^ MM,^16^^,^^43^ and CLL.^42^ The remaining 4 articles reported toxicity data for small molecule drugs used in solid tumor trials for skin,^32^^,^^48^^,^^54^ lung,^48^ esophageal,^48^ head and neck,^48^ and renal cancers,^48^^,^^50^ as well as brain metastases.^48^ In the small molecules articles where details about clinical trials were provided, there was a stronger weight toward reporting data from earlier-phase trials, with 16 phase 1 trials, 37 phase 2 trials, and 9 phase 3 trials reporting toxicity data. Additionally, 1 study reported data from a systematic review and meta-analysis of trials data as well as data from the FAERs database,^16^ so there was likely significant overlap of trials being reported across studies.

Of the 10 articles reporting small molecule therapy toxicities, all but 1 article^16^ reported sympAEs related to the use of these drugs in cancer trials. The most common sympAEs related to small molecule therapy across trials were GI, skin, and pain symptoms, as seen in Figure 3, C. The most prevalent GI symptoms reported were dysgeusia (38%-68%), decreased appetite (19%-63%), diarrhea (8%-54%), and nausea (22%-47%) with dysphonia, dysphagia, and mucositis also commonly experienced by patients. Skin symptomatic toxicities included rash (4%-68%), alopecia (38%-66%), and photosensitivity (31%), and these symptoms tended to occur more with use of TK inhibitors, BRAF inhibitors, MEK inhibitors, and HH inhibitors. Pain was also widely reported across small molecule trials with sympAEs including arthralgia (13%-42%), headache (15%-39%), myalgia (19%-22%), and pain exacerbation (17%). Other frequently reported sympAEs related to small molecule therapy included fatigue (15%-75%), nosebleeds (50%), and respiratory symptoms such as cough (23%) and dyspnea (3%).

Other clinician-reported AEs that were commonly reported across the small molecule studies included significant cardiac, respiratory, and hematological toxicities (shown in Figure 4, D), which were the most cited reasons for treatment discontinuation.^33^ Prevalence of overall cardiotoxicity was 18% with specific AEs of hypertension (3%-25%), atrial fibrillation (2%-12%), heart failure (1%-5%), and myocardial infarction (up to 15%) reported across studies. Respiratory toxicities reported were primarily pneumonia (5%-10%) and acute respiratory distress syndrome (4%), whereas hematological toxicities cited were cytopenias (5%-35%), bleeding events (5%-10%), and severe hemorrhage (3%-5%). It should be noted that these severe AEs were reported much more frequently with the use of TK inhibitors and proteosome inhibitors compared with agents with other MOAs.

*Monoclonal antibodies.* Six articles reported sympAEs related to mAbs, which were primarily used in trials to treat hematological cancers, such as MM,^16^^,^^52^ MCL,^44^ ALL,^39^ and CLL.^42^ One study reported sympAEs related to mAbs for patients with a variety of solid tumors,^48^ including glioblastoma, head and neck cancer, esophageal cancer, bladder cancer, and rectal cancer. In the mAb articles where details about clinical trials were provided, a relatively even number of early vs late-phase trials reported toxicity data, with 5 phase 1 trials, 9 phase 2 trials, and 10 phase 3 trials. Additionally, the study citing toxicities for mAbs used in solid tumor patients reported results from 23 trials that were described as being either phase 1 or phase 2.^48^

Of the 6 articles reporting toxicities related to the use of mAbs, all but 1 article^39^ reported sympAEs trials data. The most common sympAEs related to mAbs across studies were fatigue (37%-64%), diarrhea (26%-53%), nausea (35%-42%), cough (23%-40%), and mucositis (31%), with peripheral neuropathy (7%-29%) and dyspnea (15%-17%) also frequently reported (see Figure 3, D). Fever and alopecia were also reported in a few articles, but no prevalence data were provided for these symptoms. Other clinician-reported AEs that were commonly reported across the mAb studies included myelosuppression, infusion reactions (3%-51%), and cardiac toxicity. Myelosuppression was nearly universal across studies and was often severe with anemia (99%), neutropenia (12%-96%), thrombocytopenia (30%-87%), and lymphopenia (34%) reported, which likely contributed to episodes of bleeding experienced in 17% of patients. Treatment-related AEs for mAbs were reported to be 35%, with treatment-related fatal AEs between 3% and 4% (typically due to severe hematological or cardiac toxicities). Figure 4, E highlights commonly reported AEs related to mAb therapy in cancer trials.

## Discussion

This scoping review included 34 studies that reported on the prevalence of toxicities, including sympAEs, for patients taking newer cancer drugs being tested in clinical trials. To our knowledge, this is the first review to report sympAEs information across both immunotherapy and targeted therapy cancer trials, thus addressing an important gap in the literature given the rapid incorporation of these therapeutic approaches in modern cancer trials. In this section, we provide recommendations in light of our findings that may improve the quality of patient-reported sympAE data collected in cancer trials and enhance our understanding of how these drugs affect cancer patients, particularly in the early phases of drug development.

Our findings highlight a common set of leading sympAEs that are frequently reported across newer drugs used in cancer trials, regardless of target or MOA, including fatigue, diarrhea, and rash. Cancer-related fatigue has always been one of the most frequently reported symptoms by patients,^55^ even during periods of remission or surveillance, suggesting that this symptom cannot be solely attributed to treatment. This has important implications for how baseline symptoms are accounted for in cancer trials, particularly in early-phase trials where the focus is determining a new drug’s side effect profile and safety parameters.^56^ The common findings of GI and skin sympAEs reported in this review suggest that these may be core treatment-related symptoms that should be systematically collected and reported in all cancer trials, including those that are evaluating immunotherapy and targeted therapy agents.

Although there are common leading sympAEs across both immunotherapy and targeted therapies, our findings also show a different set of sympAEs within each treatment modality. For example, vomiting, fever, and itchiness were seen in at least 40% of patients being treated with immunotherapy (ICIs, T-cell therapies, IMiDs, vaccines). On the other hand, taste changes, decreased appetite, and alopecia were reported in at least 50% of patients being treated with targeted therapy (small molecules and mAbs). There also were some mechanism-specific sympAEs that, although not as prevalent, can still pose significant risks for harm and disability to patients. An example is the vision-related sympAEs that were reported associated with ICI therapy, most frequently with nivolumab, which tend to be quite rare but if not identified and treated early can result in permanent vision loss.^23^^,^^26^ Although not included in this analysis, antibody drug conjugates can also have substantive ocular toxicity that can pose safety issues and impaired functioning for trial participants.^57^ Clinical presentation of ocular symptoms include dry, itchy, or watery eyes; eye pain; and double or blurry vision, which if present warrant early involvement of an ophthalmologist to determine the diagnosis and best management to preserve vision.^23^ This relatively rare toxicity might be overlooked if basing sympAE measurement decisions purely on the most prevalent symptoms across cancer trials. Researchers and clinicians who are designing cancer trials using newer agents should include core toxicities, as well as any mechanism-specific toxicities that have been reported, to provide additional details about timing, severity, and impact of important symptoms for patients during trial participation.

An interesting finding from our review is that many studies tended to report drug toxicities as AEs or syndromes solely using the CTCAE reported by clinicians without additional self-report from patients using the PRO-CTCAE or other PROMs. This was not as apparent for the ICI review articles as a whole; however, only 36% of T-cell therapy review articles cited any sympAEs and those that did reported very few and lacked prevalence data for those symptoms. Instead, these trials reported AEs as broader syndromes, such as CRS, ICANS, and neurotoxicity, and provided prevalence data for those conditions but not for the underlying symptoms that define them.^33^^,^^35^^,^^40^ There are multiple reasons why reporting AEs solely in this way is not optimal, both from a patient safety perspective and for ensuring high-quality trials data. First, past research has consistently shown that clinicians often underreport their patients’ symptom occurrences and severity using the CTCAE,^4^^,^^58^^,^^59^ likely due to differences in grading schema for toxicities captured by clinician vs patient-reported assessments. The PRO-CTCAE was developed to allow complementary reporting of sympAEs by both clinicians and patients in order to capture an accurate portrait of the subjective experiences of patients participating in cancer trials^58^ and, importantly, may facilitate improved communication between the patient and their clinical team. Also, systematic sympAEs collection with real-time monitoring by clinicians may inform early identification of important clinical syndromes, which likely will confer a safety advantage for patients experiencing them if they receive prompt treatment. Additionally, sympAEs data obtained directly from the patient using PROMs, such as the PRO-CTCAE, can facilitate improved communication between clinicians and patients and better symptom control while on trial, and these data could be incorporated into regulatory decisions about drug approvals and labeling when considering tolerability and impact on quality of life.

We have highlighted the purpose and potential utility of collecting patient-reported sympAEs in cancer clinical trials evaluating newer therapies, but the question remains as to how best to collect these data consistently within trials, capturing toxicities common across treatments and those that are mechanism-specific, all in the least burdensome way for patients. The FDA has emphasized the need for clear designation and monitoring of treatment-related toxicities and disease-related symptoms when evaluating new therapeutic approaches.^58^^,^^60^^,^^61^ In 2014, Reeve et al.^62^ published recommendations for a core set of patient-reported symptoms to be measured in all adult cancer treatment trials, which included the following 12 symptoms: fatigue, insomnia, pain, appetite loss, dyspnea, cognitive problems, anxiety, nausea, depression, sensory neuropathy, constipation, and diarrhea. There are several symptoms in this core set that can be affected by a newer cancer drug and warrant close monitoring, such as diarrhea, nausea, and dyspnea, but there are also several symptoms listed that are more likely related to the patient’s disease and their illness trajectory over time. In early-phase trials, it may be prudent to have a shorter core list of sympAEs to capture toxicities commonly associated with cancer treatments with addition of any mechanism-specific items relevant to that particular agent, while using the write-in function of the PRO-CTCAE to capture any unanticipated events. As more data become available about contemporary cancer treatment approaches, re-evaluation of these recommendations for sympAEs monitoring may be warranted.

A few limitations to this review warrant discussion. This was a scoping review of other review articles and practice guidelines, and there are inherent limitations to this type of methodology. These include taking a more high-level approach to summarize sympAEs data from individual studies and a lack of bias assessment for included literature, compared with a systematic review approach.^63^ As such, we did not review the individual trials source data that we reported toxicity data from; rather, we abstracted trial sympAEs (and other AEs) data from the review articles summarizing those trials and were limited in what we could report based on how those data were presented. For example, some sympAEs were mentioned as being commonly associated with a particular therapy, but if no prevalence data were provided, then we were unable to quantify that symptom or give an accurate assessment of how impactful it may be for trial patients. Also, the focus of the included review articles likely influenced the sympAEs and other toxicities reported, particularly if they were most interested in symptomatic toxicities related to a particular body system or syndrome. Additionally, although we aimed to report on sympAEs captured using the PRO-CTCAE in adult cancer trials, the vast majority of data came from solely reported CTCAE data in the included literature, which may have underestimated occurrence and severity of symptoms patients experienced during trial participation. There also was significant heterogeneity in the data reviewed for this analysis, including the phase of trial, class of agent, and type of cancer, which may have affected our findings. However, we analyzed sympAEs for each class of oncological agents separately with a goal to provide a clearer picture about tolerability of those drugs and any unique toxicities that clinicians should be aware of. Last, the population for this review was focused exclusively on adult oncology patients, although we recognize that many of these oncological agents are used in pediatric patients and warrant further study.

## Conclusion

In summary, we identified several core sympAEs experienced by patients participating in clinical trials using a variety of immunotherapy and targeted therapy drugs, which has implications for future trial design and drug labeling. In addition to clinician-reported sympAEs data, systematic assessment of patient-reported sympAEs may provide complementary tolerability data that could allow better global assessment of the overall benefit vs risk ratio of emerging cancer therapies. Standardization and incorporation of these patient-reported sympAEs may improve later-phase trial designs, help support drug approval, and promote shared decision making between providers and patients to inform treatment decisions.

## Supplementary Material

pkaf036_Supplementary_Data

## Data Availability

Data sharing is not applicable to this article as no new data were created in this study.
